# Cerebrospinal fluid leakage repair of various grades developing during endoscopic transnasal transsphenoidal surgery

**DOI:** 10.1371/journal.pone.0248229

**Published:** 2021-03-26

**Authors:** Il Hwan Lee, Do Hyun Kim, Jae-Sung Park, Sin-Soo Jeun, Yong-Kil Hong, Sung Won Kim

**Affiliations:** 1 Department of Otolaryngology–Head and Neck Surgery, Seoul St. Mary’s Hospital, College of Medicine, The Catholic University of Korea, Seoul, Republic of Korea; 2 Department of Neurosurgery, Seoul St. Mary’s Hospital, College of Medicine, The Catholic University of Korea, Seoul, Republic of Korea; University of Adelaide, AUSTRALIA

## Abstract

**Objectives:**

We describe the strategy used to repair intraoperative leaks of various grades and define factors for preventing postoperative cerebrospinal fluid leakage (CSF) after surgery via the endoscopic endonasal transsphenoidal approach (EETA).

**Study design:**

Retrospective chart review at a tertiary referral center.

**Methods:**

Patients who underwent surgery via EETA from January 2009 to May 2020 were retrospectively reviewed. Intraoperative CSF leakage was graded 0–3 in terms of the dural defect size; various repairs were used depending on the grade.

**Results:**

A total of 777 patients underwent 869 operations via EETA; 609 (70.1%) experienced no intraoperative CSF leakage (grade 0) but 260 (29.9%) did. Leakage was of grade 1 in 135 cases (15.5%), grade 2 in 83 (9.6%), and grade 3 in 42 (4.8%). In 260 patients with intraoperative CSF leakage, a buttress was wedged into the sellar defect site in 178 cases (68.5%) and a pedicled flap was placed in 105 cases (40.4%). Autologous fat (108 cases, 41.5%) and a synthetic dural substitute (91 cases, 35%) were used to fill the dead space of the sellar resection cavity. Postoperative CSF leakage developed in 21 patients: 6 of grade 1, 7 of grade 2, and 8 of grade 3. Buttress placement significantly decreased postoperative leakage in grade 1 patients (p = 0.041). In patients of perioperative leakage grades 2 and 3, postoperative CSF leakage was significantly reduced only when both fat and a buttress were applied (p = 0.042 and p = 0.043, respectively).

**Conclusion:**

A buttress prevented postoperative CSF leakage in grade 1 patients; both fat and buttress were required by patients with intraoperative leakage of grades 2 and 3.

## Introduction

The endoscopic endonasal transsphenoidal approach (EETA) is safe and effective when used to remove pituitary adenomas and parasellar tumors, and is widely used worldwide [1–3]. However, it is associated with a relatively high rate of intraoperative cerebrospinal fluid (CSF) leakage and a postoperative leakage rate of 3–15.9% [4–6]. Leakage can cause meningitis attributable to ascending bacterial infection from the nasal cavity, and it can make serious sequelae. Leakage repair is essential. Esposito *et al*. [7] introduced a repair approach based on leakage grade. A smooth or rigid buttress served to minimize CSF pulsation and reduce the risk of repair failure. However, more evidence is required. We have performed graded repair for more than 10 years; in this paper, we describe risk factors for postoperative CSF leakage and the strategy that we employ when encountering intraoperative leakage.

## Materials and methods

### Patients

This study and the associated chart review were approved by the Institutional Review Board of the Catholic University of Korea, Seoul. St. Mary’s Hospital, College of Medicine (approval no. KC17RESI0354). Written informed consent was obtained from all patients and data were fully anonymized. All patients treated via EETA by our neurosurgery/otolaryngology team at Seoul St. Mary’s Hospital from January 2009 to May 2020 were retrospectively reviewed. We collected demographic data, tumor pathologies, CSF leakage grades, the methods and materials used for sellar reconstruction, repair outcomes, and previous history of surgical area irradiation.

### Surgical technique and repair of intraoperative CSF leakage

**Fig 1:**
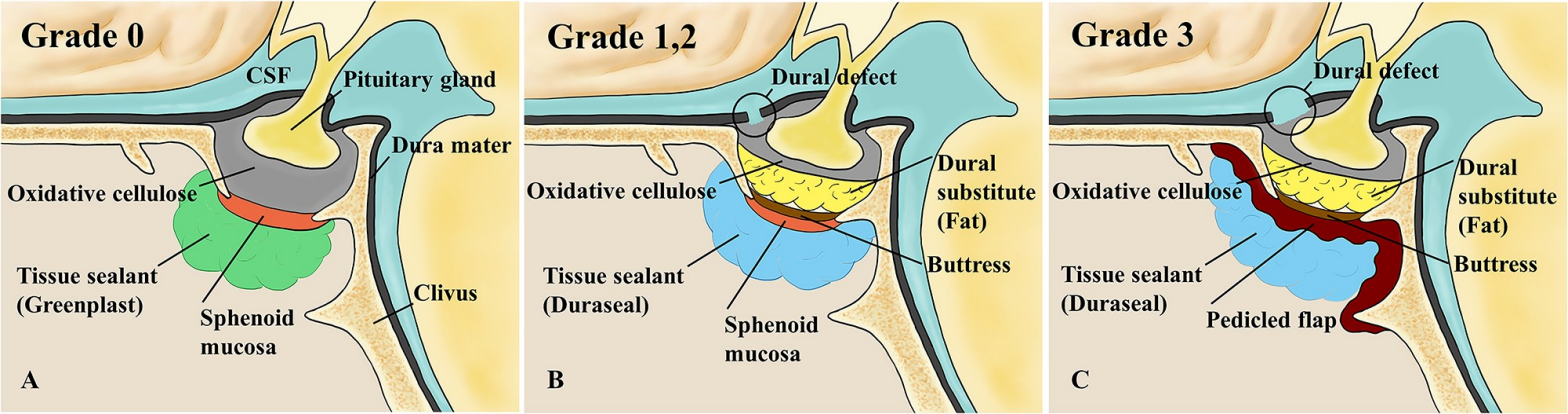
Repair of intraoperative CSF leakage by grade. (A) Grade 0. (B) Grades 1 and 2. (C) Grade 3. (Reproduced from Park et al. [10] under a CC BY license, with permission from The Korean Neurosurgical Society, original copyright, 2015.). CSF, cerebrospinal fluid.

All operations were performed using the two-nostrils/four-hands technique [8]. A rhinology surgeon harvested bilateral modified nasoseptal rescue flaps [9]. After the bony portion of the sellar floor was exposed, a neurosurgeon drilled out the floor and opened the dura mater. After removing the tumor, both surgeons reconstructed the sellar defect. The neurosurgeon performed intrasellar reconstruction and the rhinology surgeon reconstructed the outer portion of the sella. As previously reported [7], intraoperative CSF leakage was graded by reference to the size of the dural defect. Absence of leakage (as confirmed by the Valsalva maneuver) was graded 0 and a small “weeping leak” without a visible diaphragmatic defect was graded 1. Grade 2 reflected moderate leakage combined with an obvious diaphragmatic defect; grade 3 leakage was associated with a large diaphragmatic or dural defect. The neurosurgeon performed all grading; the repair method varied by grade (Fig 1).

#### Grade 0

In the absence of leakage, the neurosurgeon filled the sellar resection cavity with oxidative cellulose (Surgicel/Ethicon; Johnson & Johnson, Somerville, NJ, USA) [11] and then the rhinology surgeon repositioned (reflected) the sphenoid sinus mucosa to cover the sellar floor [12]. We were careful to not invert the mucosa because of the risk for a later mucocele. Next, more oxidative cellulose was added until the mucosa did not move. The Valsalva maneuver was performed to confirm the absence of CSF leakage, and then the sphenoid sinus was obliterated and a tissue sealant (Greenplast; Green Cross Corp., Yongin, Korea) was applied. Unused septal bone was inserted between the bilateral modified nasoseptal rescue flaps for use as a buttress should re-operation be required, and to strengthen the flaps. The sphenoid sinus was obliterated using Nasopore (Polyganics, Groningen, the Netherlands) (an absorbable packing material). Two Merocel tampons (Medtronic Xomed Surgical Products, Jacksonville, FL, USA) were packed into the bilateral nasal cavity.

#### Grade 1

If “weeping” was observed, the sellar floor was reconstructed in layers [7]. The dead space of the sellar resection cavity was obliterated with oxidative cellulose and either a synthetic dural substitute (Duraform [Codman]; Johnson & Johnson, Raynham, MA, USA) or autologous fat [13]. Harvested septal bone was wedged into the intrasellar extradural space of the bony defect. The reflected, sphenoid sinus mucosa was repositioned and more oxidative cellulose was added. A tissue sealant (DuraSeal; Covidien, Dublin, Ireland) was used to close the defect [14], followed by Nasopore/Merocel packing.

#### Grade 2

When CSF leakage was moderate, sellar defect reconstruction was similar to that described above. However, after wedging the septal bone buttress, if the reflected sphenoid sinus mucosa did not cover all of the defect, the operator removed remaining sphenoid mucosa and extended the right modified nasoseptal rescue flap anteriorly and created a right conventional nasoseptal flap [12] to cover the bony edge of the surgical defect. Oxidative cellulose was used to fix the flap, and then DuraSeal, Nasopore, and Merocel were applied.

#### Grade 3

Pedicled flaps are essential to treat patients with large diaphragmatic or dural defects [15]. In our cases, after inserting a septal bone buttress, the operator removed the reflected, sphenoid sinus musosa and created a right conventional nasoseptal flap. If that was impossible because the flap had been used during a previous operation, a left nasoseptal flap was prepared. If a bilateral nasoseptal flap was impossible because of prior flap failure or removal of the nasal septum, a middle turbinate flap was created. The pedicled flap covered the entire bony defect and was fixed with oxidative cellulose. The sphenoid sinus was obliterated with DuraSeal and Nasopore, followed by Merocel packing to ensure hemostasis.

### Postoperative care

Patients lacking intraoperative CSF leakage remained in bed only during the day of operation; the Merocel nasal packing was removed on postoperative day 3. Patients exhibiting intraoperative leakage remained in bed until postoperative day 3 and the nasal packing was removed on day 5 [16]. Lumbar drainage was not routinely used to prevent postoperative CSF leakage.

### Statistics

Numerical variables are expressed as means ± standard deviations. The chi-square and Fisher’s exact tests were used to compare categorical variables (sex, pathology, CSF leakage grade, and the reconstructive materials used). We employed binary logistic regression analyses to identify independent predictors of postoperative CSF leakage. A *p*-value < 0.05 was considered to indicate statistical significance. All statistical analyses were performed using SPSS software ver. 24.0 (IBM, NY, USA).

## Results

### Patient characteristics

A total of 777 patients (387 [49.8%] males and 390 [50.2%] females) underwent 869 EETA-facilitated operations from February 2009 to May 2020. The mean patient age was 50 ± 15.4 years (range 15–86 years). A total of 739 surgeries (85%) were primary and 130 (15%) were revisions.

### Intraoperative CSF leakage

A total of 609 operations (70.1%) were associated with no leakage; 260 (29.9%) featured leakage (grade 1 in 135 [15.5%], grade 2 in 83 [9.6%], and grade 3 in 42 [4.8%]; Table 1).

**Table 1:** Intraoperative and postoperative CSF leakage rate.

CSF leakage grade	Intraop CSF leakage (rate)	Postop CSF leakage (rate)
Grade 0	609 (70.1%)	0 (0%)
Grade 1	135 (15.5%)	6 (28.6%)
Grade 2	83 (9.6%)	7 (33.3%)
Grade 3	42 (4.8%)	8 (38.1%)
Total	869 (100%)	21 (100%)

### Reconstruction materials

In the 260 patients exhibiting intraoperative leaks, a buttress was used in 178 (68.5%) to reconstruct the skull base. Harvested septal bone was most commonly employed (175 cases); an absorbable artificial plate was used in 2 cases. We employed septal cartilage in only one case. Two types of material were used to fill the dead space of the sellar resection cavity: autologous fat was employed in 108 cases (41.5%) and Duraform (a collagen-based, biocompatible dural substitute) was used in 91 (35%). And lumbar drainage was used in 20 cases (7.7%) to divert CSF.

### Defect reconstruction using pedicled flaps

A total of 105 cases (40.4%) required pedicled flaps; we prepared 94 nasoseptal flaps (36.2%) and 11 (4.2%) middle turbinate flaps when neither a right nor left nasoseptal flap was available.

### Postoperative CSF leakage

Postoperative CSF leakage developed in 21 (2.4%; 8 males and 13 females; Table 2) of the 869 cases, thus in 8.1% of the 260 cases exhibiting intraoperative leakage. Leakage rate did not significantly differ by sex (p = 0.438). Revision operations (14.9%) were associated with more leakage than primary operations (5.7%) (p = 0.017, odds ratio [OR] = 2.903). The postoperative leakage repair failure rate (28.6%) was higher than that of intraoperative leakage repair (p = 0.003, OR = 5.973). In terms of pathology, craniopharyngiomas (36%) were associated with the highest repair failure rate (p<0.001, OR = 10.453). Pituitary adenomas were associated with a significantly lower rate of postoperative leakage (4.3%; p<0.001, OR = 0.202). Postoperative CSF leakage was of grade 1 in 6 cases (4.4%), grade 2 in 7 (8.4%), and grade 3 in 8 (19%). Grade 1 and grade 3 were significantly associated with postoperative leakage (p = 0.025, OR = 0.341 and p = 0.01, OR = 3.71, respectively). The postoperative leakage rate did not show significant differences by the reconstructive material used or by the type of flap employed. However, the reconstructive material led to a significant difference according to the leakage grade (Table 3). Of the 80 grade 1 patients with buttress, we encountered only one (1.3%) repair failure (p = 0.041, OR = 0.127). For grade 2 patients, the repair failure rates after use of a buttress (9.2%, p = 1) and fat (2.5%, p = 0.111) did not significantly differ. However, it significantly decreased when fat and a buttress were used together (0%, p = 0.042, OR = 0.592); this was also true of grade 3 patients (0%, p = 0.043, OR = 0.618).

**Table 2:** Correlation between factors and postoperative CSF leakage.

	Intraop CSF leakage	Postop CSF leakage	Rate (%)	p value	odd ratio
Gender				0.438	1.457
Male	115	8	7.0		
Female	145	13	9.0		
Age (yr)	50.4 ± 15.6	50.3 ± 15.7		0.4	1.013
Surgery					
Primary	193	11	5.7	0.017*	0.345
Revision	67	10	14.9	0.017*	2.903
Repair surgery of postop CSF leakage	21	6	28.6	0.003*	5.973
Pathology					
Pituitary adenoma	188	8	4.3	< 0.001*	0.202
Rathke’s cleft cyst	17	1	5.9	1	0.697
Craniopharyngioma	25	9	36.0	< 0.001*	10.453
Meningioma	10	3	30.0	0.576	1.278
Chordoma	9	0	0.0	1	0.962
Chondrosarcoma	1	0	0.0	1	0.992
Arachnoid cyst	1	0	0.0	1	0.992
Others	9	2	22.2	0.159	3.489
CSF leak Grade					
Grade 1	135	6	4.4	0.025*	0.341
Grade 2	83	7	8.4	0.885	1.072
Grade 3	42	8	19.0	0.01*	3.71
Material of reconstruction					
Buttress	178	12	6.7	0.232	0.578
Fat	108	5	4.6	0.086	0.413
SDS	91	10	11.0	0.206	1.773
Pedicled vasculized flap	105	12	11.4	0.12	2.021
Nasoseptal flap	94	11	11.7	0.106	2.067
Middle turbinate flap	11	1	9.1	1	1.145

*P<0.05 for the test.

SDS, synthetic dural substitute.

**Table 3:** Correlation between postoperative CSF leakage and material for reconstruction by grade.

	Intraop CSF leakage	Postop CSF leakage	Rate (%)	p value	odd ratio
Grade 1	135	6			
Buttress	80	1	1.3	0.041*	0.127
Fat	54	3	5.6	0.683	1.529
SDS	34	1	2.9	1	0.582
Fat + Buttress	33	1	3.0	1	0.606
SDS + Buttress	26	0	0.0	0.596	0.798
Grade 2	83	7			
Buttress	65	6	9.2	1	1.729
Fat	40	1	2.5	0.111	0.158
SDS	35	5	14.3	0.126	3.833
Fat + Buttress	31	0	0.0	0.042*	0.592
SDS + Buttress	30	5	16.7	0.092	5.1
Grade 3	42	8			
Buttress	33	5	15.2	0.336	0.357
Fat	14	1	7.1	0.233	0.231
SDS	22	4	18.2	1	0.889
Fat + Buttress	13	0	0.0	0.043*	0.618
SDS + Buttress	20	4	20.0	1	1.125

*P<0.05 for the test.

SDS, synthetic dural substitute.

## Discussion

### Causes of and risk factors for postoperative CSF leakage

A total of 777 patients underwent 869 operations associated with 260 intraoperative CSF leaks, and 21 postoperative leaks developed in 15 patients (Table 4). The postoperative CSF leakage rate after intraoperative CSF leakage was 8.1%; the overall rate was 2.4%. In other studies on patients with various types of tumors, the overall postoperative CSF leakage rate has been reported to be 1.6–15.9% [6, 7, 17–23]. Thus, it is important to identify the causes and perioperative risk factors for leakage when planning sellar repair.

**Table 4:** Clinical feature of 21 cases with postoperative CSF leakage.

Leak No.	Gender	Age	Pathology	Surgery	Leak grade	Systemic disease	Smoking	RT	Pedicled flap	Dural substitute	Buttress	POD	Meningitis	Treatment
1	M	52	Pituitary adenoma	Primary	2	(-)	(-)	(-)	(-)	SDS	SB	14	(+)	Revise repair
2	F	77	Pituitary adenoma	Revision	2	HTN	(-)	(-)	NSF	SDS	SB	3	(+)	Revise repair
3	F	77	Pituitary adenoma	Revision	2	HTN	(-)	(-)	NSF	SDS	SB	6	(+)	Revise repair
4	F	44	Meningioma	Primary	1	(-)	(-)	(-)	(-)	SDS	(-)	12	(-)	Revise repair
5	F	28	Pituitary adenoma	Revision	2	NF	(-)	(-)	(-)	SDS	SC	6	(-)	Revise repair
6	M	57	Pituitary adenoma	Revision	3	(-)	(-)	(+)	(-)	SDS	SB	7	(+)	Revise repair
7	M	57	Pituitary adenoma	Revision	3	(-)	(-)	(+)	NSF	SDS	SB	101	(+)	Revise repair
8	M	31	Craniopharyngioma	Primary	3	(-)	(+)	(-)	NSF	SDS	SB	26	(+)	Revise repair
9	M	33	Craniopharyngioma	Revision	3	(-)	(-)	(+)	NSF	SDS	SB	12	(-)	Revise repair
10	F	60	Epidermoid cyst	Primary	3	(-)	(-)	(-)	NSF	(-)	SB	25	(+)	Revise repair
11	M	32	Craniopharyngioma	Primary	2	(-)	(+)	(-)	(-)	(-)	SB	6	(-)	Revise repair
12	F	28	Craniopharyngioma	Primary	3	(-)	(-)	(-)	NSF	(-)	(-)	10	(+)	Revise repair
13	F	59	Pituitary adenoma	Revision	1	HTN	(-)	(-)	MTF	Fat	(-)	3	(+)	Revise repair
14	F	56	Craniopharyngioma	Revision	1	(-)	(-)	(-)	(-)	Fat	(-)	8	(-)	Revise repair
15	F	56	Craniopharyngioma	Revision	2	(-)	(-)	(-)	NSF	Fat	(-)	20	(-)	Revise repair
16	F	56	Craniopharyngioma	Revision	1	(-)	(-)	(-)	(-)	Fat	SB	12	(-)	Revise repair
17	F	53	Pituitary adenoma	Primary	1	(-)	(-)	(-)	(-)	(-)	(-)	4	(-)	Revise repair
18	F	53	Pituitary adenoma	Primary	2	(-)	(-)	(-)	NSF	SDS	SB	14	(+)	Revise repair
19	M	79	Craniopharyngioma	Primary	3	DM,CVD	(+)	(-)	NSF	(-)	(-)	4	(+)	Revise repair
20	M	79	Craniopharyngioma	Revision	3	DM,CVD	(+)	(-)	NSF	Fat	(-)	5	(+)	Revise repair
21	F	50	Rathke’s cleft cyst	Primary	1	(-)	(-)	(-)	(-)	(-)	(-)	5	(+)	Revise repair

HTN, hypertension; NF, neurofibromatosis; CVD, cardiovascular disease; NSF, nasoseptal flap; MTF, middle turbinate flap; SDS, synthetic dural substitute; SB, septal bone (vomer); SC, septal cartilage.

Mucosalization is compromised when the recovered sphenoid mucosa or a pedicled flap becomes detached around the defect site. Flap detachment may reflect necrosis caused by partial tearing during surgery or pedicle damage caused by a hot endoscope. Detachment may also reflect poor resistance to leakage pressure. Joen *et al*. described 14 repair failures; fascial graft disruption was evident in 5 cases treated using a multilayered non-vascularized technique; these cases had a lack of counter-pressure [24]. The margins of the sphenoid mucosa and pedicled flaps are initially nourished via plasmatic diffusion from the surrounding mucosa. If the margin is detached before a surrounding vascular network forms, marginal attachment is weakened, creating a passage for CSF leakage within the first 3 to 4 postoperative days.

We sought factors associated with postoperative CSF leakage. Of the pathological factors, craniopharyngiomas have been associated with particularly high CSF leakage rates (3.3–23.4%) compared to those of other tumor types [7, 25, 26]. Our rate was 28.1%. It is difficult to completely resect craniopharyngiomas. It is necessary to open the sellar floor widely, and it is difficult to wedge a buttress into such a large defect. In addition, craniopharyngiomas often compress the floor of the third ventricle and exhibit intraventricular extensions. Opening of the third ventricle during surgery increases both CSF leakage and CSF pressure [2]. Most leakage is of grade 3; the postoperative leakage rate is high. Our intraoperative CSF leak rate in craniopharyngioma patients was 78.1% (grade 3 in 56%). In addition, tumors are often adherent to surrounding tissue because of recurrence, or prior radiation therapy. Thus, the postoperative CSF leakage rate is high in craniopharyngioma patients (p<0.001, OR = 10.453).

Operative revision and repair of initial postoperative CSF leakage significantly increased the risk of subsequent leakage (by 14.9%, p = 0.017 and 28.6%, p = 0.003, respectively). Septal bone (particularly the vomer) had often been removed or used for reconstruction during the primary operation, and thus could not serve as a new buttress, increasing the risk of subsequent leakage. If the septal bone is not required for a buttress during primary operation because no CSF leakage was encountered, it is important to store remaining bone between the septal mucosa for use (if necessary) during re-operation.

Age, smoking history, systemic disease such as diabetes mellitus (DM) or cardiovascular disease (CVD), and prior irradiation of the head and neck are known risk factors for pedicled flap failure because they compromise the flap blood supply [27, 28]. Advanced age (>60 years) is usually associated with changes in the vascular system, particularly arterial structure and function; however, we found no significant relationship between age and the postoperative CSF leakage rate (p = 0.40) [29]. Of patients who experienced postoperative leakage, three were smokers, two had DM, and three had CVD including hypertension. Two had undergone prior radiation therapy. Smoking causes vasoconstriction; DM triggers microvascular disease and thrombus formation inducing vascular obstruction [30]. CVD can damage vessel walls. Hypertension is a chronic systemic disease caused by functional and structural macrovascular/microvascular changes that compromise tissue perfusion and cause ischemia [31, 32]. Arterial and venous irradiation trigger perivascular fibrosis, endothelial damage, and microvascular occlusion [33]. Zanation *et al*. found that 2 of 16 patients with postoperative CSF leakage had undergone preoperative radiation therapy; such therapy tended to increase the postoperative leakage rate [18]. The risk factors act together to increase the risk of flap failure.

### Repair of low-grade intraoperative CSF leakage

Most leakage during surgery was of grade 1 (51.9%). Two principal methods have been used to repair such leaks. Wang *et al*. repaired sellar defects using only a gelatin sponge and a hydrogel sealant overlay. Of 74 patients treated, 2 developed postoperative CSF leakage [34]. Kelly *et al*. repaired the sellar defect employing a single layer of collagen sponge or autologous fat followed by application of a rigid or semirigid buttress such as a titanium mesh, septal bone, or a synthetic material [6, 7]; the postoperative leakage rate was only 1.9% [6]. We have used both methods and encountered six cases of postoperative leakage in grade 1 patients. The 80 patients for whom buttresses were placed exhibited significantly less postoperative leakage than did the 55 patients for whom buttresses were not placed (p = 0.041). Regardless of whether fat or a synthetic dural substitute was used, a buttress adequately countered CSF pressure in grade 1 patients. The pressure was displaced by the buttress; the sphenoid mucosa did not become detached.

### Repair of high-grade intraoperative CSF leakage

In cases with grade 2 intraoperative CSF leakage and the sphenoid mucosa does not cover the sellar defect, and in grade 3 cases, pedicled flaps counter the high CSF flow. Hadad *et al*. used a pedicled nasoseptal flap to reconstruct skull base defects [15]. It is very difficult to render such reconstruction watertight if only the extradural layer is repaired, because the CSF is derived from the subarachnoid space. A pedicled nasoseptal flap is not a stand-alone repair; careful reconstruction of each compartment is required to repair the defect [2]. Hadad *et al*. used a multilayer technique employing an inlay of collagen matrix and an onlay of an additional fascia graft or autologous fat, but did not use a buttress [15]. We inlay autologous fat (43.2%) or a synthetic dural substitute (45.6%) in almost all patients with grade 2 or 3 leakage, and often also place a buttress (78.4%). As shown in Table 4, buttress, fat, and the synthetic dural substitute were not significantly associated with postoperative CSF leakage in grade 2 and 3 patients. However, leakage was significantly reduced when both a buttress and fat were used for repair (p = 0.042 and p = 0.043 for grade 2 and 3 patients, respectively).

Both autologous fat and a synthetic dural substitute are widely used for dead space obliteration and watertight closure of the dura mater; they also divert the CSF pressure. However, if the intracranial pressure rises and the CSF pressure thus also increases, the dural substitute may be pushed back to the sphenoid sinus. This risk increases if the dural defect is large, as in patients with grade 2 and 3 leakage, raising the risk of postoperative leakage. The buttress not only diverts the CSF pressure but also prevents dural substitute migration; the effects of the two materials are synergistic. Fat persists for longer than synthetic dural substitutes such as Duraform; adipose tissue cells exhibit a regenerative capacity fueled by nutrient diffusion from surrounding tissue [13]. We found that postoperative CSF leakage was significantly reduced when fat (rather than a synthetic dural substitute) was combined with a buttress.

### Buttress for CSF leakage repair

Various materials may serve as buttresses; the vomer portion of the septal bone is most commonly used. The vomer is simple to harvest during posterior septectomy, and is easy to shape with scissors. However, the maximal possible amount of vomer should be harvested and carefully shaped by reference to the defect size. Septal cartilage can also serve as a buttress; this is simple to harvest but is not rigid. Thus, cartilage may slip if not fixed. One of our patients underwent revision EETA-associated surgery to treat a pituitary adenoma; grade 2 intraoperative CSF leakage developed. Septal cartilage was used as a buttress because the vomer was absent. However, the cartilage slipped, and was pushed out by CSF pressure on postoperative day 6; the nasoseptal flap became detached and postoperative CSF leakage occurred. Therefore, if the septal bone has been removed during prior surgery, the use of an artificial buttress should be considered. Of the various artificial buttresses, titanium mesh and a polyethylene plate (Medpor Tsi Barrier; Stryker CMF, Kalamazoo, MI, USA) are often used. A rigid absorbable plate (TnR Mesh TSI; T&R Biofab Co. Ltd., Korea) served as the buttress for two of our patients. The plate can be easily cut and shaped. The buttress is larger than that afforded by septal bone; the material can be used for repair after removal of craniopharyngiomas and other tumors via wide openings in the sellar floor.

### Limitations

Sine our study is a retrospective nature, the results are not as definitive as those of randomized controlled studies. All sellar floor with same leakage grade did not be reconstructed with same method. However, we reconstructed most of sellar defect in the way introduced previously. And this allowed for comparison of the occurrence of postoperative CSF leakage according to the reconstruction material in the same leakage grade.

## Conclusion

Both a buttress and a dural substitute have been thought to be valuable for sellar floor reconstruction. In this study, we confirm that both significantly prevented postoperative CSF leakage. If leakage is encountered intraoperatively, sellar defect reconstruction must consider the leakage grade, the pathology, and patient condition; postoperative complications must be minimized.

## Supporting information

S1 Table Data of 260 patients with intraoperative CSF leakage. (XLSX)
